# Critical Faculty and Peer Instructor Development: Core Components for Building Inclusive STEM Programs in Higher Education

**DOI:** 10.3389/fpsyg.2022.754233

**Published:** 2022-05-30

**Authors:** Claudia von Vacano, Michael Ruiz, Renee Starowicz, Seyi Olojo, Arlyn Y. Moreno Luna, Evan Muzzall, Rodolfo Mendoza-Denton, David J. Harding

**Affiliations:** ^1^D-Lab, University of California, Berkeley, Berkeley, CA, United States; ^2^Department of Psychology, University of California, Berkeley, Berkeley, CA, United States; ^3^School of Information, University of California, Berkeley, Berkeley, CA, United States; ^4^Graduate School of Education, University of California, Berkeley, Berkeley, CA, United States; ^5^Department of Sociology, University of California, Berkeley, Berkeley, CA, United States

**Keywords:** faculty development, stem, culturally responsive teaching, teacher professional development, peer to peer, multicultural education, liberation pedagogy

## Abstract

First-generation college students and those from ethnic groups such as African Americans, Latinx, Native Americans, or Indigenous Peoples in the United States are less likely to pursue STEM-related professions. How might we develop conceptual and methodological approaches to understand instructional differences between various undergraduate STEM programs that contribute to racial and social class disparities in psychological indicators of academic success such as learning orientations and engagement? Within social psychology, research has focused mainly on student-level mechanisms surrounding threat, motivation, and identity. A largely parallel literature in sociology, meanwhile, has taken a more institutional and critical approach to inequalities in STEM education, pointing to the macro level historical, cultural, and structural roots of those inequalities. In this paper, we bridge these two perspectives by focusing on *critical faculty and peer instructor development* as targets for inclusive STEM education. These practices, especially when deployed together, have the potential to disrupt the unseen but powerful historical forces that perpetuate STEM inequalities, while also positively affecting student-level proximate factors, especially for historically marginalized students.

## Introduction

The challenges to effectively serving students from groups historically underserved in STEM are deep and longstanding. A long line of research shows that first-generation college students, as well as Black, Indigenous, People of Color (BIPOC) students, often lose interest after their initial engagement with STEM education at the college level. Research has identified multiple obstacles that these students face in STEM educational environments, including feelings of isolation due to the low numbers of their close peers in STEM, gaps in preparation, and experiences with structural and interpersonal prejudice within STEM programs (Seymour and Hewitt, 1997; Barr et al., 2008; Vanasupa et al., 2009; Smith et al., 2015; Harackiewicz et al., 2016; Harrington et al., 2016; Killpack and Melón, 2016; McGee, 2016; Aikens et al., 2017; Eastman et al., 2017; Farrell and Minerick, 2018).

Social psychology and sociology approach the understanding of these obstacles from different perspectives. Social psychology, by and large, has historically focused on the individual-level mechanisms and psychological processes that affect student engagement, belonging, identification, and achievement. Such *student*-level analyses generally have not focused on the *macro* level processes that sociological approaches emphasize: the ways in which historically rooted, gender, race, and class-informed structures of power create and exacerbate inequalities in STEM education.

Both student and macro perspectives are crucial to understanding and reducing inequalities in STEM education, but because they approach their analyses from different vantage points, their implications for intervention – that is, for reducing disparities – can appear at odds, and difficult to integrate. One of our goals in this paper is to bridge the student and macro perspectives specifically with respect to the development and evaluation of intervention. We strive to connect the institutional and organizational context not only to the historical and cultural forces, but to the individual-level experience, with the objective of designing interventions to bring about systemic change. We do so by focusing on interventions at the *meso*-level; that is, the systems through which macro level influences are translated to individuals within communities (Serpa and Ferreira, 2019). Meso level structures include both formal and informal systems that organize social groups, and include both physical structures (e.g., banks and schools) as well as “ways of doing” (e.g., curricula, promotion, and hiring processes). More specifically, we focus on *critical faculty and peer instructor development* as two separable but related meso level practices through which to effect change in the broader ecology of STEM education. Both practices, we argue, can positively impact minoritized students’ achievement and belonging, while also shining a light and disrupting some of the otherwise invisible macro level influences that contribute to inequities in STEM education.

Broadly, critical approaches to pedagogy encompass a group of meta-reflective practices that encourage instructors to actively recognize the hidden influences, assumptions, and norms that govern instruction and learning (e.g., López and Burciaga, 2014; Ladson-Billings and Tate, 2016). These approaches depart from traditional pedagogical training in that they do not focus so much on content *per se*, but rather, on the historical and sociological forces that act upon the practice of pedagogy. Critical approaches to pedagogy openly invite analysis and reflection of topics traditionally not considered in STEM training, including history, discrimination, power dynamics, and one’s own positionality within the learning environment. Here we discuss how critical approaches can be used as an essential tool for faculty development in STEM, and we also explore how critical approaches can help inform the *intentional* training of peer instructors. Intentional peer instruction refers to education by peer instructors in a structured or scaffolded way, and stands in contrast from student-initiated informal peer instruction (e.g., getting help from a friend on homework). This approach aims to make sure that peer instructors learn and grow themselves, at the same time that they are helping or assisting their peers.

**FIGURE 1:**
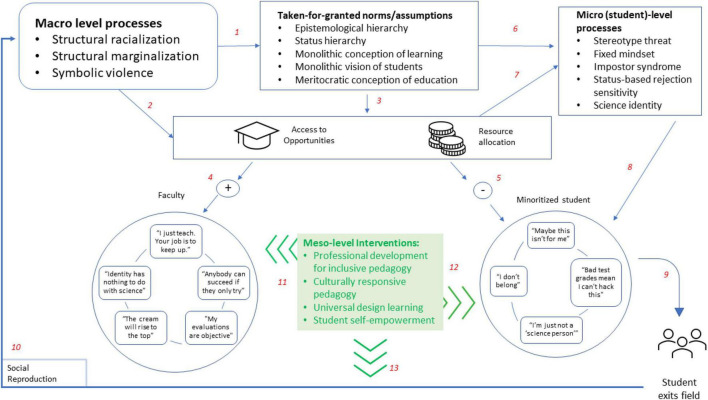
Relationships between micro, meso, and macro level factors.

Figure 1 presents a schematic representation of how these practices interrupt an otherwise cyclical process. The two large circles in the bottom half of the figure represent a faculty member and a minoritized student, each with a set of thoughts or cognitions that might represent their thought processes in a traditional STEM course or program. Given that the majority of STEM faculty are white and male (Bennett et al., 2020), we conceptualize the faculty member as possessing these characteristics. Above the faculty member and the student are the constructs that we have outlined here. In the top left corner, we list the broad and historically influenced macro level processes that then shape both the taken-for-granted norms and assumptions of the traditional educational system (arrow 1), as well as the behavioral manifestations of these processes relating to access and resource allocation (arrow 2). Arrow 3 acknowledges that the system norms facilitate decisions about access and resources, which tend to favor the people who have historically ended up as faculty (4) but disfavor minoritized students (5). Both the assumptions and the disparities in access and resources create the environment for the student level processes we summarize here (6 and 7), which then affect the student’s thought process and decision-making (8). These processes have been linked to disparities in completion and retention that affect minoritized students most severely (9), which contributes to social reproduction of the macro level conditions (10) that feed the recursive system.

In the center of the figure, in green, are the meso level interventions we outline below. The green arrows stemming from the green box are specifically interruptive processes within the otherwise recursive system. The pedagogical interventions provide an opportunity to change the cognitions and attitudes of the faculty member (11), as well as of the student (12). The interventions interrupt social reproduction processes (13), and ultimately, we hope, have an effect on the macro processes themselves by increasing access and representation in the field.

Our central argument is that meso level interventions that include a critical approach to faculty and peer instructor development may be key to addressing STEM disparities, given that meso level processes play a mediational role between macro and student-level factors. To illustrate the usefulness of this approach, we select and discuss a few prominent factors identified in the psychological and sociological literatures to further elucidate some of the macro and student-level constructs in the figure. Our aim here being illustrative rather than encyclopedic, we do not aim for a comprehensive review of all the student-level or macro level processes that ultimately affect student achievement. Similarly, at the meso level, we focus on a few examples of interventions relevant to student achievement. Throughout this process we have been informed by interviews and focus groups conducted with students in Data Science at UC Berkeley; their experiences and insights both inform and shape the recommendations we present here. We present data from these qualitative sources to give voice to the challenges and opportunities that minoritized students face in the academy. In addition, we present an illustrative case study from a program at UC Berkeley to demonstrate both the promise and the challenges of interventions at this level. While a single program cannot hope to be representative of the entire landscape of various STEM programs in a wide array of heterogeneous institutional settings, the Berkeley program is discussed here for illustrative purposes.

## Student-Level Constructs

The micro or student-level perspective, rooted in social and educational psychology, tries to elucidate the psychological processes that are broadly relevant to student motivation and achievement. Disparities are explained through the differential processes that affect marginalized or stigmatized students relative to non-minoritized students. As such, the currency of this approach is the motivational, perceptual, and attitudinal factors that influence achievement. By implication, intervention from this perspective involves targeting (i.e., changing) the psychology of the student.

Leveraging findings from psychological interventions, we briefly discuss six key student-level constructs that are central to understanding the psychological experience of minoritized students in STEM fields: sense of belonging, growth mindset, imposter syndrome, stereotype threat, status-based rejection sensitivity, and scientific identity. We focus on these constructs because prior interventions demonstrate that these six factors interactively contribute to lack of persistence in STEM among historically marginalized students. We view the constructs below as interrelated and these relationships as being part of a broader constellation of constructs that are used to understand the student experience.

### Sense of Belonging

Research has shown that when historically marginalized students experience adversity throughout any segment of their student career, they are more likely to interpret these experiences as indicators of not fitting in socially or academically (Walton and Cohen, 2007). A sense of belonging has been shown to increase social and academic fit in college, and to decrease the propensity to view adversity as proof of not fitting in Walton and Cohen (2007). A sense of belonging has been tied to behaviors that include more time studying, sending emails to professors, attending office hours, fewer visits to the doctor, improved health and happiness, and more “outgroup” friends (Walton and Cohen, 2007; Walton et al., 2015).

Moreover, a sense of belonging improves students’ evaluation of their “in group” as it pertains to STEM performance and increases persistence in STEM enrollment (Walton et al., 2015; Murphy et al., 2020). Lastly, feelings of belonging in college also impact the future development of students’ professional careers and well-being such that they report greater satisfaction and success in their careers, involvement in leadership roles, and life satisfaction (Brady et al., 2020).

### Growth Mindset

Meanwhile, some people believe that intelligence is fixed, i.e., that one has a certain level of inborn intelligence that does not change through environmental input. However, there is a compelling body of research that shows that the belief that people’s intelligence can be developed through dedication and hard work (that is, grow one’s intelligence), the meaning of failure is transformed away from a diagnostic tool of one’s capacity to an opportunity to learn from one’s mistakes (Dweck and Yeager, 2019). When marginalized students hold a growth mindset, they are resilient to the effects of stereotype threat and earn higher grade point averages (Aronson et al., 2002).

### Imposter Syndrome

Imposter syndrome can be defined as a collection of feelings of inadequacy that persist despite evident success (Clance and Imes, 1978). “Imposters” suffer from a chronic sense of intellectual fraudulence that overrides feelings of success or external proof of their competence. Marginalized students are likely to confront imposter syndrome, in which they contend with feelings of doubt regarding whether they have earned their success (Clance and Imes, 1978; Canning et al., 2019a). Moreover, imposter feelings are heightened for first-generation students when they perceive their classroom environments to be competitive. This is associated with lower levels of class engagement, attendance, grades, and greater dropout intentions (Canning et al., 2019a).

### Stereotype Threat

An additional barrier that marginalized students face is stereotype threat, a phenomenon in which individuals become aware of and or are nervous about confirming a negative stereotype about their group in a domain where they are subject to negative evaluation (Steele, 1997). Stereotype threat has been shown to affect academic performance through task disengagement and domain disidentification (the former being constrained to a given academic task, the latter being more generalized such that one disengages self-esteem from the domain) (Spencer et al., 1999; Walton and Spencer, 2009; Mello et al., 2012; Casad and Bryant, 2016; Martiny and Nikitin, 2019).

### Status-Based Rejection Sensitivity

The model of status-based rejection sensitivity proposes that beyond concerns about being evaluated in light of negative stereotypes, marginalized students are likely to experience and be concerned about exclusion, marginalization, and ostracism due to their social identity. In other words, status-based rejection sensitivity recognizes the social and interpersonal implications of stigmatized status that go beyond stereotypes *per se* (i.e., people just don’t like you because of a specific characteristic). The construct of status-based rejection sensitivity has been applied to a number of different identities that include race, gender, social class, appearance, sexual orientation, age, and weight (see Mendoza-Denton and Leitner, 2018). The academic impact of status-based rejection sensitivity is mediated through the social processes through which education occurs: concerns about being rejected, for example, leading to avoidance of professors’ office hours, decreased trust in the university, and greater anxiety about belonging (Mendoza-Denton et al., 2002). Additionally, Mendoza-Denton et al. (2002) found that African status-based rejection sensitivity among African Americans was related to declines in grade point average over the course of five semesters at a Predominantly White University.

### Science Identity

According to Chen et al. (2021), students hold a strong science identity when they and their important reference groups consider them to be a “science person.” Past research has demonstrated that minority students’ self-reported scientific identity is positively associated with intentions to socially integrate into the scientific community, participate in conducting research, and apply to graduate school above and beyond the effects of self-efficacy and endorsing the science community values (Estrada et al., 2011). Woodcock et al. (2012) found that both Latinx and African American students demonstrated a positive association between scientific identity and intentions to pursue a science career (Woodcock et al., 2012). More recent research has found science identity to be positively associated with higher performance in gateway STEM classes (Chen et al., 2021), and to be a protective factor against academic underperformance for minoritized students in particular. To further elucidate the impact of science identity on performance, Chen et al. (2021) conducted a social belonging intervention that demonstrated that a sense of belonging untethered the effect of science identity from performance.

## Macro Perspectives

The above student-level constructs offer a granularized view into the proximate social forces that affect student outcomes. In introducing the macro literature, we begin by briefly describing three of the most commonly used theoretical frameworks and then discuss key macro constructs that inform our understanding of the educational climate experienced by students from marginalized groups. We then discuss how these theoretical approaches can translate into pedagogical practice.

### Theoretical Frameworks for Understanding Macro Level Inequalities

Critical race theory (CRT), a theoretical tradition rooted in African American and emancipatory thought, offers helpful insights into how issues of belongingness are racially motivated and the solutions that can be used in addressing them. CRT explores the conditions under which anti-blackness is manifested, and takes the explicit stance of naming the violence of anti-blackness across temporal, ideological and political contexts (Crenshaw, 2011). CRT was originally developed to critique legal scholarship (Caldwell, 1996), and Ladson-Billings and Tate (2016) extend CRT to higher education. They draw connections between systemic iterations of racism to stratification within higher education, showing how racism is deeply ingrained within the American educational system through its assumptions, cultural ideals, and day-to-day practices.

Another theoretical approach to addressing marginalization is post-colonial theory. Within this theoretical approach, the histories of colonial oppression are traced to their present-day impact on institutional logics and practices. This approach recognizes that the history of the American university is deeply embedded within the history of colonialism in the United States. Positioned as a frontier for western empiricism, institutions of higher education were founded on the premise of a need for religious and often agricultural expertise. Such college qualifications for many years exclusively elevated white men into America’s burgeoning middle class. From this came the development of curricula and classrooms that were centered around Whiteness (Kliebard, 2004). The deep resistance that efforts toward integration were met with laid the groundwork for the continued otherization of minoritized students within the higher education institutions (López and Burciaga, 2014).

Indigenous ways of knowing offer us an epistemological alternative to the current paradigm in which we imagine STEM instruction. While it can often expand beyond western categories of knowledge, indigenous scholars have positioned these practices as important interventions for decolonizing the academy. Sisseton-Wahpeton Oyate scholar, TallBear (2019), proposes a theory of *relationality* that prioritizes an ethic of recognition beyond social hierarchies that are often rooted in a racist and imperial past. In recognition of a colonial history of dehumanizing people and things that differ from whiteness, indigenous traditions of honoring kin have helped combat logics of otherness that have justified the marginalization of minoritized people. Métis scholar Max Liberion’s practice of citational justice illustrates how local knowledge can be preserved within the academe (Liboiron, 2021) through the art of expliciting and repeatedly acknowledging genealogies that have not commonly been recognized within academic citational practices. Liberion shows us the necessity of acknowledging the diverse avenues of knowledge production can occur, which often is not limited to pedigree and established authority. Furthermore, we see such an ethic of care as both integral to indigenous ways of knowing and emancipatory within the classroom because of its explicit recognition of agency and a shared mutual investment in power among teachers and students alike.

Similar to Indigenous scholarship, approaches like critical race theory and post-colonial theory focus their analytical attention on deconstructing structural systems rather than individuals, urging us to focus critique on systems of oppression. These frameworks emphasize that the exclusion of members of stigmatized and marginalized groups from land, resources, capital, and status is not a function of the simple aggregation of individual discriminatory behaviors, but rather is a feature of how institutions work – their internal logics, norms, and daily practices. Institutions carry forward past injustices, harms, and power relations in ways that are not easily discernible precisely because they are built into the very fabric of how the institution functions. Bourdieu and Passeron (1990), for example, develop the concept of symbolic violence, which refers to non-physical violence manifested in the difference in power relations between social groups. Symbolic violence is unconsciously agreed upon by the dominant and dominated. Norms are imposed by the group possessing greater social power over subordinate group members.

### Higher Education Institutions Through a Macro Lens

One way to understand structural and systemic inequality within the university is through what Powell (2013) calls structural racialization or structural marginalization. He positions these forms of marginalization as processes, rather than as acute events, “that may generate disparities or depress life outcomes without any racist actors” (Powell, 2013, p. 4). More importantly, with this definition, Powell prompts an analysis of “the genesis and formation of critical structures, not just how a structure operates or how programs are administered” (ibid). Embedded within these institutions are a series of financial, cultural and meritorical practices that codify the marginalization of underrepresented students.

Critical theories also understand the university as a potential site of social reproduction rather than social mobility or opportunity. Social reproduction refers to reproducing social classes in order to maintain and reify social hierarchies. Students who are born into working, middle and upper classes are led to stay within the same class as adults. Class reproduction, poverty, and unequal educational outcomes for low-income students are maintained by the role “sorting machines” that school plays (Willis, 1981; Bowles and Gintis, 2002). Although initially focused on primary and secondary schooling as institutions of class reproduction, the social reproduction perspective can be extended to post-secondary institutions. With the advent of neoliberalism, globalized and corporatized universities become more selective, and students with higher economic mobility have better chances of admission into competitive schools. These students also have the resources to maintain their standing within these institutions (through material means that afford access to housing, nourishment, and technology, for example) and receive “good quality” assistance from hired help. Social reproduction allows for educational spaces to maintain social and economic inequalities by inhibiting the social mobility of marginalized populations (Bourdieu and Passeron, 1990). From this perspective, rather than being gateways to social mobility, schools make valuable resources and coursework available to students with higher socioeconomic statuses, further marginalizing and maintaining the class positions of students.

### Implications for Curriculum and Pedagogy in STEM Education

Critical Race Theory, Post-Colonial Theory, and other macro perspectives posit multiple broad influences on STEM education that affect its participants – both instructors and learners – deeply yet invisibly, by guiding and constraining the choices, opportunities, and psychologies of individual actors. Importantly, as these processes are historical, cultural, and institutional, their influences are invisible – that is, they are “baked in” to the structure of the system in such a way that to be able to engage in the system, one must necessarily accept (or go along with) the logics and norms mandated by these processes.

These macro level influences on curriculum and pedagogy in STEM Education can be understood as a set of taken-for-granted assumptions about learning and effective teaching practices. As in the micro level section above, the discussion below is not exhaustive, but provides a blueprint for the kinds of factors that the perspective highlights.

#### Epistemological Hierarchy

Derived from European models of scientific knowledge and epistemology, this conception privileges quantifiable and seemingly objective practices of scientific measurement and explanation at the expense of knowledge derived from deep and long standing personal experiences. In implicitly or explicitly rejecting such ways of knowing and the information they can provide to science as anecdotal or unscientific, such conceptions can devalue the personal experiences of first-generation students and students of color as secondary, even when studying topics where they have direct lived experiences.

#### Status Hierarchy

Long standing, unspoken, and institutionalized notions of status and authority within the university and its classrooms can render students as passive recipients of faculty knowledge and expertise, rather than as co-creators of the learning environment. Such taken-for-granted power hierarchies disempower students and shield faculty from criticism or the need for reflection, responsiveness, or self-awareness.

#### Monolithic Conception of Learning

This conception assumes that all students learn in the same way, and that conventional or traditional modes of instruction and design of assignments and labs continue to be the most effective methods. Such a notion can be attached to the instructor’s own prior experience as a student and what was effective for them, despite scientific evidence that other methods may be more effective for a larger and wider range of students. Closely linked to this notion are ideas about what sorts of assessments are both fair and effective, such as a reliance on high-stakes tests.

#### Monolithic Vision of “the Student”

This understanding derives from the assumption that the normative college student is white and comes from a middle class background. Curricula and pedagogy are designed around strategies, timelines, and expectations that have typically worked well with that population of students in the past. A related notion is that students who differ in their racial, income, and educational background must conform to white, middle class notions of a student, rather than the instructor and institution adapting to the increasing diversity on college campuses.

#### Conception of Education as Meritocratic

A central assumption of traditional educational systems is that anybody, by dint of hard work, can achieve the highest level of success. By implication, a lack of achievement reflects on the individual’s own talents or abilities. This worldview protects against critical examination, and is fundamentally incompatible with, the recognition of the structural processes implicated in systematic oppression.

## The Meso Level

In this section, we turn our attention to the meso level that links the student-level proximate outcomes and the macro level influences to the contexts in which instruction takes place. We conceptualize the meso level as active: the pedagogical practices through which educators socialize students, model and enforce norms, and transmit the cultural standards of the macro level. This conceptualization is based on research that has demonstrated the ways in which the macro factors can influence specific behavioral responses of instructors in the meso level and how the meso level can be redesigned to encourage behaviors that result in student academic persistence (Mendoza-Denton and Mischel, 2007; Stephens et al., 2012).

The active quality of this level, we argue, provides a potential target for intervention. We focus on two specific groups of practices: critical faculty development for inclusive pedagogy, and intentional peer instruction. These pedagogies move away from deficit-oriented thinking, highlighting the learning context’s role in affecting student engagement, and the development of dialogic education.

As we elaborate and illustrate the value of inclusive teaching practices, we draw examples from our ongoing survey and qualitative research on the experiences of first-generation and BIPOC students in the UC Berkeley data science program (see Supplementary Appendix A for more information). The survey data offer insight on the factors relating to student success in data science and their continuation in this field (persistence), as moderated by the supports provided by components in the model. Within this larger correlational investigation, we embed qualitative focus groups to gain a deeper understanding of students’ experiences and the processes they deem important that contribute to their progress (or lack thereof) in their STEM experiences. The qualitative and survey studies were approved by the UC Berkeley Institutional Review Board.

To understand the relationships from the quantitative data, a subset of underrepresented undergraduate students was selected to participate in four focus groups. This qualitative data allows us to explore the experiences from the students’ perspective and the factors that students feel to be significant to their success and persistence. These qualitative data reveal the subjective perceptions and detailed experiences that are not knowable from the survey data alone. An important goal of the focus groups is also to understand the nature of the intellectual community experienced by students from underrepresented groups.

We emphasize that the data presented below are not intended to either test specific hypotheses derived from the framework or evaluate the Berkeley program. Rather, we present these data to help illustrate how the ideas, practices, and constructs we discuss are experienced in the day-to-day educational journeys of students.

### Berkeley Data Science as an Illustrative Case Study

Data science is a new STEM field, one with great potential to engage students historically marginalized in STEM through its use of social data and emphasis on social impact. Research suggests that students from diverse backgrounds often find a decontextualized model of science learning frustrating and that it too frequently fails to address the social relevance that many underrepresented students find motivating (Roundtable on Data Science Post-Secondary Education, 2017). By contrast, intellectual pursuits framed around altruistic goals—that is, goals focused on the greater good—are often strong motivators that are personally and professionally meaningful to students, particularly those who otherwise do not persist or are not retained in the sciences (Thoman et al., 2015).

UC Berkeley’s Data Science Undergraduate Studies within the Division of Computing, Data Science, and Society (Adhikari et al., 2021) employs an interrelated set of pedagogical and institutional strategies that attempt to address the linked challenges of diversity and scale and to serve undergraduates from both STEM and non-STEM majors. Berkeley’s program includes instructional Modules that “push in” data science into the existing curriculum, a zero-prerequisite Foundations course currently taken by more than 1500 students per semester, concurrent Connector courses that delve into substantive data science applications, a Data Scholars program to support students traditionally underserved in STEM, and Discovery Projects that provide students the opportunity to apply data science skills in real-world settings. The program has also recently developed both a data science major and a data science minor. Formal course curricula are enhanced by a larger ecosystem of support programs as well as student-led extra-curricular activities centered around the application of data science in multiple substantive domains. Students in the program are offered a buffet of opportunities and options to self-select into allowing for flexibility in a broad range of points of contact.

While Berkeley’s Data Science program is a nationally recognized model that is being adapted and refined by many other colleges and universities (National Academies of Sciences, Engineering, and Medicine, 2018), it nevertheless faces many of the challenges with diverse and broad STEM education faced that are felt by programs around the country, including retention of scholars, broad access, and scalability (Xu, 2016; Tuthill and Berestecky, 2017). The data presented below illustrate the ways that students experience such challenges and inform our recommendations for meso-level interventions.

### Faculty Development for Inclusive Pedagogy

An inclusive pedagogy STEM faculty development can focus on reflection, empathy, and awareness of social relations and interactions in the learning environment. These issues cut across various educational approaches, with a common focus on the constant critical analysis of the macro level processes affecting learning.

#### Reflexivity

Normally used to refer to the research process, reflexivity involves reflecting on the process of knowledge creation and the ways that our own perceptions shape everything that we see (Brownlee et al., 2017). Therefore, an examination of one’s own beliefs, judgments and practices both in the research but also in the instructional process can enable faculty to question taken for granted assumptions. In relation to this it also creates opportunities to more critically engage with diverse student identities. By articulating syllabi and curriculum that meaningfully incorporate and engage with diverse scholarship, students are provided with a more enriching educational experience. For example, the Cite Black Women movement (Smith et al., 2021) encourages multiple principles of engagement and inclusion of Black scholarship in coursework.

Central to this work is an exploration of the implicit biases that one possesses regarding their students, which stem from macro level factors like status hierarchy, a monolithic vision of the student, and conception of education as meritocratic. Thinking of a student as being a member of a minority group, being underprepared, having a disability, or being a second language learner is a different perspective from realizing that collectively minoritized groups have power and voice. Indigenous ways of knowing foreground the expertise that students derive from their daily lives and collective community experience and wisdom. For example, being disabled can mean that students bring a unique perspective to the materials. Being multilingual means understanding problems from multiple vantage points. Students come to the classroom with a variety of experiences and perspectives that can be leveraged in the work if they are encouraged and supported in doing so. However, we know that deficit perspectives, such as those derived from epistemological hierarchy, status hierarchy, and the monolithic conception of learning, are linked to lower expectations and further marginalization (Ash et al., 2020).

These claims are empirically supported by prior research. For example, when first generation students heard stories from panelists about how their social class backgrounds presented them with unique challenges in college and the strength to overcome those challenges, this resulted in a reduction in the achievement gap between first and continuing generation students, an effect that was mediated by increases in students’ tendencies to seek college resources (Stephens et al., 2014). A follow up study found that the first generation students in the intervention condition were more likely to discuss their backgrounds when giving a speech and demonstrated higher physiological thriving after a stressful evaluative task in comparison to first generation students in the control condition (Stephens et al., 2015). More recent research has also demonstrated that endorsement of lower socio-economic status students’ backgrounds is positively correlated with their academic achievement (Hernandez et al., 2021).

Research on the impact of faculty growth mindsets provides another empirical example. STEM professors’ fixed (compared to growth) mindsets about student intelligence was significantly associated with decreases in student grades, motivation, perception of faculty as emphasizing learning and development, and recommendation of their courses to other students (Canning et al., 2019a). Moreover, courses taught by STEM professors who held fixed rather than growth mindsets exhibited achievement gaps for underrepresented (Black, Latino, and Native American) students in comparison to White and Asian students (Canning et al., 2019b).

Consider also the following example, in which students of color felt excluded and othered by textbook examples used to illustrate statistical concepts.

*“There are a lot of examples in the textbook where it*… *reiterates, like stereotypes that many people face, many cultures face. So there was one about the first project talking about the world population and poverty rates in certain cultures and countries. And not a lot of people liked that since they felt like it was just emphasizing the stereotypes of many of the people, and they just didn’t feel like many people would understand their concerns since they themselves don’t have to deal with that in their day to day lives. Yeah, so that was a challenge, basing the material and having to suck up your own feelings and just do the work.”*

As this example demonstrates, pedagogy needs to reflect on the impact that particular examples or scenarios may have on students.

#### Empathy

Building relationships with students requires understanding their backgrounds, and their educational voyage, along with their ways of thinking and understanding the world around them. Creating community building opportunities outside the classroom can be a powerful way to connect with a diverse student body. Co-constructing third spaces can help students come out of their shell and lower their anxieties about the university context, potentially countering imposter syndrome and stereotype threat and promoting a sense of belonging and science identity. At the same time, these activities can build empathy on the part of faculty and other instructors by themselves learning about the historical and social context of the students they are teaching (Moll et al., 1992).

Killpack and Melón (2016) argue that this change needs to occur alongside changes in institutional culture. They provide evidence that faculty development opportunities limit discussion to comfortable topics and miss opportunities for deeper issues to be explored such as faculty privilege, implicit bias, and cues for stereotype threat. These areas of bias engender discrimination and become pervasive among faculty, instructors, undergraduate student instructors, TA’s, and section leaders.

### Awareness of Social Relations and Interactions in Classroom Learning Environments

Countering feelings of threat and exclusion at the level of the individual, as well as fostering a sense of belonging and inclusivity, requires attention to daily micro level interactions. Language and tone are key to creating inclusive classroom environments that welcome diverse groups of students and model constructive peer norms.

The idea of a safe space originates from Lesbian, Gay, Bisexual, Transexual, Queer (LGBTQ) spaces intended to bring attention to consciousness raising in the women’s movement, in which efforts have been made historically to co-construct spaces were marginalized communities could find and build community, empowerment, and resistance to social oppression. These spaces were not free from internal disagreements, but instead were characterized by shared commitments to social change. Faculty can start building a safer space by examining their own identity in relationship to the students’ identities and to the topic being taught. They can also practice having conversations that are sensitive. This can begin with establishing norms of conduct for the class that apply to both instructors and student peers. Norms of conduct can begin with an acknowledgment of the inherent issues of bias and power in data and the effort to make these issues more transparent. Faculty can welcome reflection on the part of the students and invite their thoughtful and constructive critique and can explicitly welcome student voices from every perspective, every age, race/ethnicity, national origin, immigration status, level of experience, gender, gender identity, gender expression, sexual orientation, range of abilities, or physical appearance.

Faculty can play an important role in establishing peer norms in the classroom. When pro-diversity norms are made salient in STEM classrooms (by showing students a brief video that discussed how their peers value diversity and enjoy getting to know students from different social groups) the achievement gap between privileged and marginalized students narrowed, whereas the achievement gap persisted in STEM classes assigned to the control condition (Murrar et al., 2020).

Awareness of social relations and interactions in the learning environments is also the type of knowledge, skills and disposition that faculty can develop and then foment among their graduate and undergraduate instructors, teaching assistants, and section leaders. Such efforts implicitly involve surfacing unspoken macro level assumptions like epistemological hierarchy, status hierarchy, monolithic conception of learning, monolithic vision of the student, and a conception of education as meritocratic. Diversity education reduces race-related biases among participants and can help build bias reduction strategies (Devine and Ash, 2022). Faculty from medicine and STEM departments involved in these types of faculty development have increased their personal awareness of biases, increased motivation and belief in their ability to promote equity. When at least 25% of a department’s faculty were involved in gender bias training, participants reported statistically significant increases in their personal efforts to promote gender equity (Carnes et al., 2015). These studies shed light on the potential for positive change through these faculty development programs provided that there is broad participation.

### Culturally Responsive Pedagogy

Culturally responsive pedagogy is defined as using the cultural characteristics, experiences, and perspectives of ethnically diverse students as conduits for teaching students more effectively (Kleinfeld, 1975; Au and Kawakami, 1994; Ladson-Billings, 1994, 1995; Foster, 1995; Gay, 2000). Both Freire (1968) and Hooks (2014) also imagined liberatory pedagogies that center questions of agency and power within the classroom as ways that counter epistemological and status hierarchies.

When the curriculum is not closely related to students, it can result in alienation from their sense of knowing and from education broadly (Au, 2012). With culturally responsive teaching comes the acknowledgment or reclaiming of the curriculum. Versions of history that were previously invisible or illegible within classrooms can come to light (Au et al., 2016). Black feminist epistemologies of thought have historically centered knowledge building practices around the personal and affective experiences of the individual (Collins, 2002). Also, Indigenous knowledge practices have challenged western empiricism and prioritized local knowledge practices that are contextually and geographically sensitive (Kimmerer, 2013; Coulthard, 2014; Smith et al., 2015). The reclamation of multicultural history, voices and research enriches the classroom. Diverse histories and ways of knowing need to be incorporated into the curriculum and there needs to be acknowledgment that these epistemologies and ontologies are essential for advancing contemporary thought on campuses. There is mounting evidence that instructional congruence has a positive effect on learners’ ability to assimilate knowledge (Lee and Fradd, 2001; Cuevas et al., 2005). However, this approach requires an extensive amount of commitment, including reflection upon the instructor’s identity, culture and language and match or mismatch to students’ own identity, culture, and language.

The value of cultural congruence is illustrated by a student who preferred to learn data science ethics in a department where they expected greater congruence with their own identities and experiences. The focus group students agreed and shared their preference to wait for an ethics course to be offered in the African American Studies Department that would meet the curricular requirement. The students felt that this specific course would most closely align with their interests and was worth the wait for an irregular offering rather than taking the standard Data Science ethics course.

We acknowledge and understand that professors already manage a complex set of formal and informal roles and responsibilities, such as serving as research leaders, upholding standards of scholarship, influencing public debate, representing their department and university, serving on university committees, and generally being a role model. However, in this paper we want to highlight the importance of faculty as critics, advocates, and intellectual leaders who are vested in social and political concerns. Data science is a rich ground to develop efforts for social good and social justice, whether related to the incarceration system, refugee crisis, houselessness, health, or the environment, etc. Expectations of professors are implied and there is increasing agreement that faculty members, like any other professional, need guidance and development and there are indications that there is very little provision of these (Macfarlane, 2012).

### Universal Design for Learning

The effort to articulate materials in diverse ways for student reception and the acceptance of multiple forms of expression is the basis of Universal Design for Learning (UDL) (Meyer et al., 2014). Universal Design for Learning is an educational framework based on research in the learning sciences, including cognitive neuroscience that guides the development of flexible learning environments and learning spaces that can accommodate individual learning differences. It is also consistent with an ethic of care that prioritizes meeting all students where they are. Students in our focus groups discussed self-knowledge about their preferences for particular forms of curricular materials (project-based vs. tests) and its impact on their learning. They shared awareness about how the course design has a direct impact on their success. We highlight the opportunity of UDL in the design and ongoing iterative implementation of courses that invite and engage student voices in ever-changing technosocial spheres.

For example, one student in our focus groups highlighted concerns with trying to understand and navigate the multiple avenues of communication and activity that a course uses. She shared her confusion and concerns, which eventually led to the development of a chart that would clearly outline each of the web based spaces, providing access and defining the use for each. This facilitated her understanding of what she was expected to be doing and how she was to access these course resources. This was not an initial part of classroom planning but certainly provides a valuable resource that could be built in from the start of coursework design. It allows for both explicit access and demonstrates multiple means of participation where students can demonstrate their knowledge and share questions. Other students shared that near-peer programs offered well designed and executed programming to help them build skills at crucial moments with accessible content, meeting their learning needs at their current point in their learning trajectory.

Universal Design for Learning provides multiple facets of instructional design that open up a variety of avenues for students to engage receptively and expressively with contant. These mechanisms break down previously assumed epistemological and status hierarchies of instructional format and encourage courses that are dynamic and iterative in order to enhance the teaching and learning experiences of instructors and students, thereby promoting sense of belonging, growth mindset, and science identity while countering imposter syndrome, stereotype threat, and status-based rejection sensitivity. This work has been taken up and expanded in close relationship to Critical Race Theory by Fritzgerald and Rice (2020). This effort potentially can interrupt stereotypes and bias in curricular materials and lectures by acknowledging and critiquing power differentials that frequently can manifest in microaggressions and institutional violence.

### The Role of Peer Instructors in Supporting Students From Various Backgrounds

As faculty develop their own inclusive pedagogical practices, they can in turn model and transmit these same practices to peer instructors through discussion and mentorship. Consistent with several scholars’ conceptualizations (Talbot et al., 2015; Vickrey et al., 2015; Schell and Butler, 2018), we view an important part of near-peer instruction as facilitating peer group discussions independent of the larger lecture course that is led by a faculty instructor. This is because interactions between peers, unmediated by faculty, produce important learning gains for both peer learners and peer instructors (Secomb, 2008; Evans and Cuffe, 2009; Talbot et al., 2015; Balta et al., 2017).

Peers can have a unique and powerful influence in the context of instruction, as students may see peer instructors as more relatable, less threatening, and easier to approach. Nonetheless, peer-to-peer dynamics are not immune from the dynamics of prejudice and exclusion that can characterize faculty-student interaction. In the following section, we review some of the benefits – as well as the challenges – that students of color experienced in the context of peer instruction.

Building on the literature on third spaces that welcomes multiple scripts into a space of interaction and engagement focused on learning (Gutierrez et al., 1997), students in our focus groups appreciated the ability to come with the subject in dynamic workshops and in an environment where any question was welcome. Even more powerful are self-organized groups, like a group of Black engineering students at UC Berkeley who call themselves the “C.S. mob.” They have created organic peer-to-peer networks that became nets of information sharing and support. A student elaborated:

*I do think that I genuinely gravitate towards people who look like me, either like ethnic minorities or women. I think that there’s just a sense of comfortability, as well as seeing someone who looks like you. I have a friend who said, honestly, “(Person’s name), if you didn’t reach out to me while we were taking 61A, I don’t think I’d be studying CS anymore.” And, being able to. Want to work with us or if they have any questions. Maybe they know what they’re doing and maybe they can help my friends and then go to classes and meet other people from very similar backgrounds to you. Just having that sort of group build.*

This student went on to discuss how the organization looks for other students to invite in. The student discusses how with any given question they feel comfortable reaching out, knowing that someone will reach back and support them in solving problems. These forms of student self-organized groups bring together critical elements of student self-determination with resources to build a supportive data science education framework. The need for students to build their own groups stems from their collective effort to build educational spaces that resolve the challenges that they experience within the current education framework. Continuing to investigate how these organizations connect with departmental and curricular networks to utilize and leverage resources to their networks can provide models for encouraging other groups to build and sustain such practices.

Many students also described collaborative group projects as better aligned with their learning styles, especially when they provide opportunities to work with students from similar backgrounds and are appropriately supported by peer leaders. One student remarked:

*“I do wanna say that I’m happy that at least my project, 3 out of the 5 students are in the Data Science Scholars because we get to see each other like on the side and like we have our group meetings. So, like just with the Data Scholar students, so it’s kinda like team building, like we all get to talk as a group so it does make me feel more comfortable with the students. Whereas if I didn’t have these facilitated, like team-bonding type of things, it would have been a lot harder to communicate and express ourselves with each other. And I do think it helps that there are students that are in the Data Scholars program because we all come from different backgrounds that are more similar to students that aren’t in the program. And I think one thing that helps too is that our lead is pretty helpful.”*

A strength and a challenge of the UC Berkeley program is the use of scalable near-peer instruction with teams of undergraduates who support peer learning and co-create course materials. These peer instructors apply their learning and thereby advance their own understanding and skills. Students from our qualitative research shared the critical role of these instructors as it relates to completing course assignments. They described the variability in instructional practices, noting it was difficult in office hours to go through multiple instructors providing minimal support until finally finding a peer instructor who would work closely with them through multiple-step questions:

*“…the third person was like, ‘oh well okay. Well let’s actually delete all of this and rework it out and let’s go step by step.’ And it’s the fact that it took like three people to help me in that process, like that’s ridiculous.”*

We also learned of other difficulties in peer-instruction spaces. One example included a student noting exclusion in these spaces remains, noting it was “not as friendly. Like it was supposed to be a collaborative session where you worked with your partner, and again, it’s just really hard to find a group of people who would want to work with you in those sections.” Students provided examples where they had issues finding a project partner or having peers turn and look away from them during activities, so as not to end up as partners with them. Students across focus groups brought up concerns about the lack of diversity in the peer instructors, difficulties being able to access these employment opportunities, and the necessity for these roles to be filled with individuals who understand teaching the content from diverse points of entry. Also, it is challenging for a peer to provide mentoring if they themselves have not been mentored. We know from research that mentorship is rare and marginalized students rarely receive mentorship in higher education (Trujillo et al., 2015).

### Recommendations for Reforming Peer Instruction

Research on peer instruction has typically been conducted in college contexts that are predominantly white, and the benefits of this pedagogical approach are still unclear in more diverse colleges (Vickrey et al., 2015). Research is needed to understand the effects of peer instruction in racially diverse college contexts on racial minority students’ learning. Developing peer instruction programs requires attention to research that shows that peer interactions can *either* ameliorate *or* exacerbate the effects of stereotype threat on racially diverse students’ learning outcomes and subsequent degree progress (Taylor and Walton, 2011; Leslie et al., 2015; Storage et al., 2016; Murrar et al., 2020).

We suggest that peer instruction can be improved by using findings from the growth mindset, social belonging, and future identity literatures to provide structured guidelines that leverage the unique social influence of each student to cultivate learning environments that minimize the effects of stereotype threat (Wilson, 2011; Destin et al., 2018). We focus on both the growth mindset and social belonging literature because the effects of these interventions have been replicated throughout the United States and have been shown to reduce the racial achievement gaps in regards to end of term grades. We also include the future identity literature because it demonstrates promising effects for underrepresented students who might feel they are stereotyped as only being able to obtain low status and or non-science based jobs and therefore disengage from academic spaces or efforts to obtain upward social mobility. In the following sections, we will discuss suggestions for reforming peer instruction with an emphasis on the ways that peer instructors can leverage research results when developing their pedagogical approach. These are not intended to be comprehensive, but rather provide some examples of the types of interventions that are expected to improve student learning and retention based on prior research.

### Peer Instructors Can Encourage Growth Mindsets

When near-peer instructors are reviewing concepts from a faculty lecture, they can emphasize that some concepts might require more effort to learn and provide their peers with examples of different study strategies. Moreover, as near-peer instructors guide their peers in discussing concepts with one another, they can encourage students to discuss the strategies that they used to overcome challenges in learning the concepts. This group discussion strategy removes the negative effects of the stereotype that racially diverse students may face regarding their groups’ intellectual ability by placing emphasis on learning requiring effort and not innate ability, that is, by cultivating growth mindsets (Aronson et al., 2002). This approach to leading peer discussion will result in peer group members feeling less imposter syndrome, more trust and commitment toward the group, and perceive others as more collaborative (Canning et al., 2020; Muradoglu et al., 2021). These findings are important for peer learning because such approaches empower students to encourage one another to develop growth mindsets, in turn creating a learning environment that explicitly discourages stereotypes.

### Peer Instructors Can Cultivate a Welcoming Environment

When instructors first begin meeting with their peers, they can lead their students through group exercises that create a pro-diversity norm and reframe adversity as both common and surmountable. We highlight two such exercises, validated in the research literature. One is a brief norm setting exercise that might include everyone in the class sharing with one another one or two reasons why they value diversity. The research suggests that activities like this can help underrepresented students perceive a more inclusive and welcoming environment while non-minority students will notice this pro-diversity norm and tune their behavior to display more friendly behaviors toward their peers (Murrar et al., 2020). A second exercise includes everyone discussing a challenge that they have experienced at one point or another in their college careers and explaining how they were able to overcome it. The research has demonstrated that such discussions prevent challenges from being interpreted as indicators of lack of belonging (Binning et al., 2020). Such exercises are significant because underrepresented students have come to expect that they will be rejected in educational spaces by their non-minority peers, faculty, and campus administration due to racial stigma (Mendoza-Denton and Leitner, 2018). These exercises are likely to encourage racial minority students to feel more comfortable when engaging with non-minority peers in discussion groups because they will not be as concerned with race-based rejection (Martiny and Nikitin, 2019). These two brief exercises can substantially improve the traditional peer instruction model by creating a context that values diversity and views challenges as surmountable.

### Peers Instructors Can Nurture Their Peers’ Possible Future Scientific Selves

The negative stereotypes that racially minority students confront regarding their groups’ intellectual abilities likely limit the types of careers that they envision for themselves. As a result, such students may be less likely to engage with science education than their white peers or to be motivated to persist in the face of adversity. Near-peer instructors are in a unique position to assist their peers with developing an understanding of the science careers that they might consider. This can be done through discussions on career options and by discussing how to reach those careers through education. These peer group discussions will likely benefit all students, but especially racial minority students who are still developing their plans for science education and science careers (Oyserman et al., 2006; Destin and Hernandez, 2020).

## Conclusion

Parallel literatures in Psychology and Sociology have historically approached inequities in higher education from different angles. Psychology, with its focus on the individual, has tended to focus on student-level mechanisms that affect motivation and performance. Examples of such mechanisms that we have presented here center around feelings of threat, identity, and belonging. Sociology, with its focus on social organizations and institutions, has tended to focus on structural forces at work in the development and maintenance of inequities. Such a focus has given rise to critical approaches from which concepts such as structural marginalization and structural racism arise.

We have proposed here that interventions aimed at the meso level, directed toward those who teach and mentor students (both faculty and peers), are likely to be effective and long-lasting because they can acknowledge and act both on macro level and student-level processes. Many of the ideas we propose, including empathy, reflection, culturally responsive pedagogies, and Universal Design for Learning, encourage educators to move beyond the “sage on the stage” model of top-down learning (Mendoza-Denton, 2019), and instead reflect critically on their positionalities and the ways that students’ own backgrounds and knowledge influence learning. Peer-to-peer learning can also help break down hierarchical barriers; however, programs must be intentional to make sure that peer instructors reap benefits and professional development opportunities from their own efforts to help their peers. We have presented an example of the Data Science Program at Berkeley as an illustration of the tensions and challenges that can arise from one attempt to implement a program at the meso level. Our ultimate contention here is that instructors need to engage in a critical exploration of not only many bodies of knowledge, and ways of knowing, but also the political structure of the university, and the higher education classroom, including the various technologies associated with current teaching practices.

## Data Availability Statement

The datasets presented in this article are not readily available because we are unable to share study data due to IRB protections. Requests to access the datasets should be directed to DH, dharding@berkeley.edu.

## Ethics Statement

The studies involving human participants were reviewed and approved by UC Berkeley Committee for the Protection of Human Subjects. The patients/participants provided their written informed consent to participate in this study.

## Author Contributions

CV, RM-D, and DH contributed to conception and design of the study. CV, MR, RS, SO, AM, and RM-D reviewed the literature. CV, RS, and SO collected and analyzed the qualitative data. AL and EM performed statistical analysis. CV wrote the first draft of the manuscript. All authors contributed to the development of the theoretical framework, wrote sections of the manuscript, revised the manuscript, read, and approved the submitted version.

## Conflict of Interest

The authors declare that the research was conducted in the absence of any commercial or financial relationships that could be construed as a potential conflict of interest.

## Publisher’s Note

All claims expressed in this article are solely those of the authors and do not necessarily represent those of their affiliated organizations, or those of the publisher, the editors and the reviewers. Any product that may be evaluated in this article, or claim that may be made by its manufacturer, is not guaranteed or endorsed by the publisher.
