# Supplementary material for: Critical Faculty and Peer Instructor Development: Core Components for Building Inclusive STEM Programs in Higher Education
Source: Front Psychol. 2022 May 30;13:754233. doi: 10.3389/fpsyg.2022.754233 (PMC9197167; doi:10.3389/fpsyg.2022.754233)
Supplement: Supplementary file 1 [file Data_Sheet_1.docx]

**Supplemental Materials for**

**“Critical faculty and peer instructor development: core components for building inclusive STEM programs in higher education”**

Claudia von Vacano, Michael Ruiz, Renee Starowicz, Seyi Olojo, Arlyn Y. Moreno Luna,

Evan Muzzall, Rodolfo Mendoza-Denton and David J. Harding

**Appendix A: Methodology and Results from the Berkeley Data Science Case Study**

*Qualitative Focus Groups*

Four focus groups of undergraduate data science students from this project provided excerpts to illustrate specific components. A protocol of questions was developed by the research team that was used to guide the conversations. Topics from this protocol include: defining what data science means to the group, the students’ experiences with data science and their future interests in data science. The meetings were each facilitated by one member of the research team who was culturally, racially/ethnically, and gender matched to the focus group. Each of the four focus groups was made up of students from diverse backgrounds who were invited to participate in the study directly by one of the research team members. Focus groups had from four to six participants. The meetings were conducted on Zoom and video recorded. Each focus group ran for approximately 90 minutes. Participants were provided with a 25 dollar e-gift card for their participation. Recorded videos were used to develop transcripts that were imported into MaxQDA and coded based on previously defined codes and with codes emerging from details of the conversations.

We used the Yin method of cyclical coding (Yin, 2009) combining inductive and deductive methods. The qualitative team was trained using various analytic techniques drawing from Miles and Huberman (1994) to analyze patterns and to identify and categorize themes. We went through three different cycles and clustered themes and discarded codes that had little data. We did not conduct inter-rater reliability at this stage, but several team members coded the same data and we met to discuss our interpretations. Each focus group followed the same semi-structured interview protocol. The focus groups were composed of different types of students (Black, Latinx, transfer, female identified, male identified, non-marginalized students). The focus group facilitator belonged to the identity group that was interviewed, in order to have a cultural match and build strong rapport. The data are only being used as examples here, and we are not using the data to substantiate our claims.

*Surveys*

Students were surveyed on various dimensions regarding their self-efficacy, attitude, scientific identity, and sense of belonging in STEM. Collectively, this information offers insight on how participating in the various learning experiences can contribute to students’ motivation and strategies for learning in STEM, their engagement in STEM courses in comparison to the data science foundations course, and their sense of being part of a community that could affect plans for future STEM course taking and careers. To measure these dimensions, we rely on previously vetted surveys including, but not limited to, Activation/Engagement (Moore et al., 2011), Motivational Strategies and Learning Questionnaire (Pintrich et al., 1991), and Sense of Belonging Scale (Hurtado & Carter, 1997). Student surveys also allowed us to collect data on intentions for graduate school and careers in STEM.

We began by investigating two survey datasets to better understand undergraduate student experiences between three different ethnic groups at the University of California, Berkeley: BIPOC (a pooled group including Black, Latinx, Southeast Asian, and Native American respondents), Asian, and White. While we recognize that East Asian students are also students of color, we distinguish them from Latinx, Indigenous, and Black students because they exhibit different patterns and outcomes. The first survey consisted of the MyExperience survey (<https://myexperience.berkeley.edu/>), administered to the entire UC Berkeley campus community to learn about participation of its various students, faculty, and staff by focusing on 19 questions divided into 4 blocks: Climate, Exclusion, Institution, and Respect. The second survey dataset consisted of the Improving Undergraduate Student Education survey, subsetted for this analysis to include only students who were enrolled in the Data8: Foundations of Data Science course (<http://data8.org/zero-to-data-8/intro.html>) at the end of the Spring 2020, Fall 2020, or Spring 2021 semesters. This second survey consisted of 81 questions divided into 13 blocks: Belonging, Community, Discrimination and Exclusion, Ethnic Tension, Fascination, Growth Mindset, Identity, Informed, Preparation, Relationship, Self Beliefs, Setting, and Support. Statistically significant differences are based on one-way analysis of variance with Tukey test for Honest Significant Difference corrections for multiple comparisons at p < 0.05. An asterisk above the Asian and/or White bars in the figures below indicates a statistically significant difference to the BIPOC group.

Analysis of MyExperience survey data from n = 724 student respondents across seven disciplines (Applied Mathematics, Cognitive Science, Economics, Integrative Biology, L&S Computer Science, L&S Data Science, and Statistics) demonstrates that BIPOC students felt statistically significantly less supported by faculty and the institution as a whole (Figure A1). According to these data and compared to Asian and White ethnic groups, BIPOC students felt less comfortable with campus, departmental, and classroom climates and also that diversity, equity, and inclusion were less valued in these spaces (Climate). They also experienced more exclusionary and bullying behaviors, (Exclusion), feel less valued by faculty, the Chancellor, Vice Chancellors, Deans, and other leadership (Institution), and also that their ethnicity, socioeconomic statuses, gender identities, religious beliefs, and incarceration histories are less respected on campus (Respect).


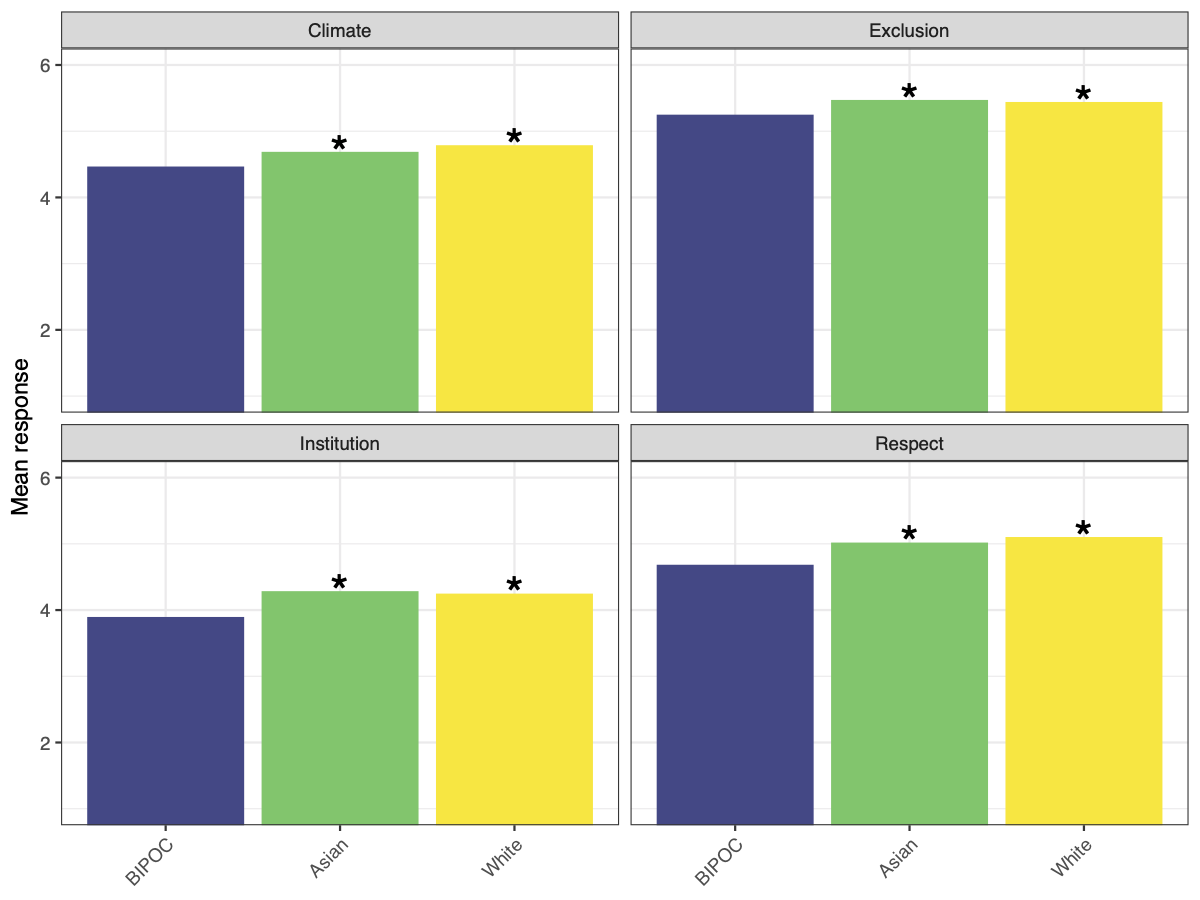


Figure A1. Mean responses from the MyExperience survey dataset for three ethnic groups BIPOC, Asian, and White based on n = 724 respondents across seven disciplines: Applied Mathematics, Cognitive Science, Economics, Integrative Biology, L&S Computer Science, L&S Data Science, and Statistics.

When considering only the L&S Data Science major (Figure A2), BIPOC students still had statistically significantly lower mean responses for Climate and Institution, while Exclusion and Respect were not statistically significantly different. Unfortunately however, the sample for this major only consisted of n = 40 respondents (n = 3 respondents for BIPOC, n = 32 for Asian, and n = 5 for White groups).


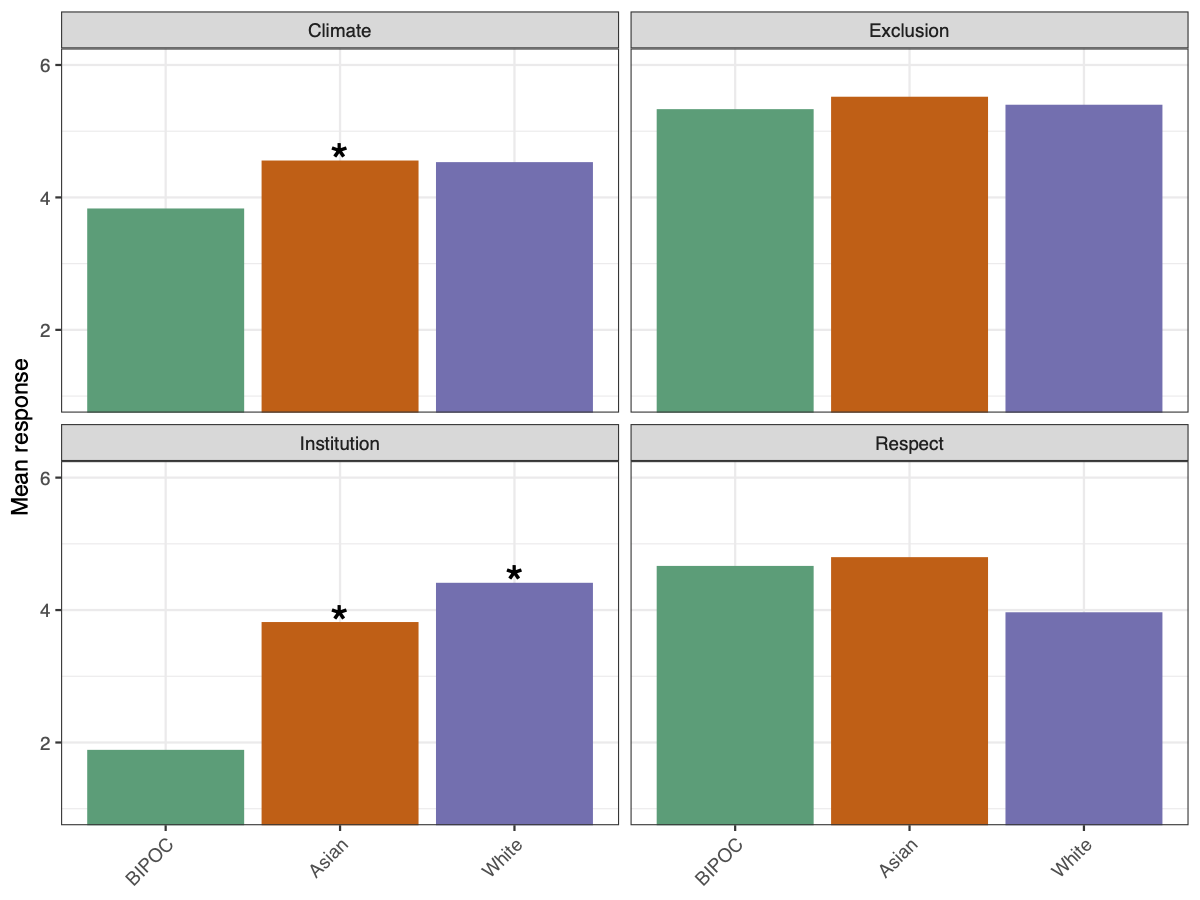


Figure A2. Mean responses from the MyExperience survey dataset for three ethnic groups BIPOC, Asian, and White based on n = 40 respondents within the L&S Data Science major.

Analysis of the Improving Undergraduate Student Education survey data from n = 286 respondents from the Data8 course (end of term) suggests that BIPOC students face many of these similar challenges as highlighted by the MyExperience survey including significantly lower mean responses compared to their Asian classmates for Belonging, Fascination, Identity, Preparation, and Self Beliefs and to their White classmates for Community, Relationship to science, Setting, and Support (Figure A3). Furthermore, BIPOC students report statistically significantly lower mean responses for Community, Setting, and Support compared to their Asian and White classmates *and* significantly higher mean responses for Discrimination and Exclusion and Ethnic Tension. However, BIPOC students show a greater sense of belonging compared to their White counterparts along with the highest mean responses for Growth Mindset which is statistically significantly greater than the White group. Furthermore, BIPOC students have statistically significantly higher mean responses for Belonging compared to the White group, and comparable scores for Informed, Preparation, and Self Beliefs. These results might suggest that BIPOC students are just as confident and able as their Asian and White classmates but face significantly greater institutional barriers that are likely harmful to success.


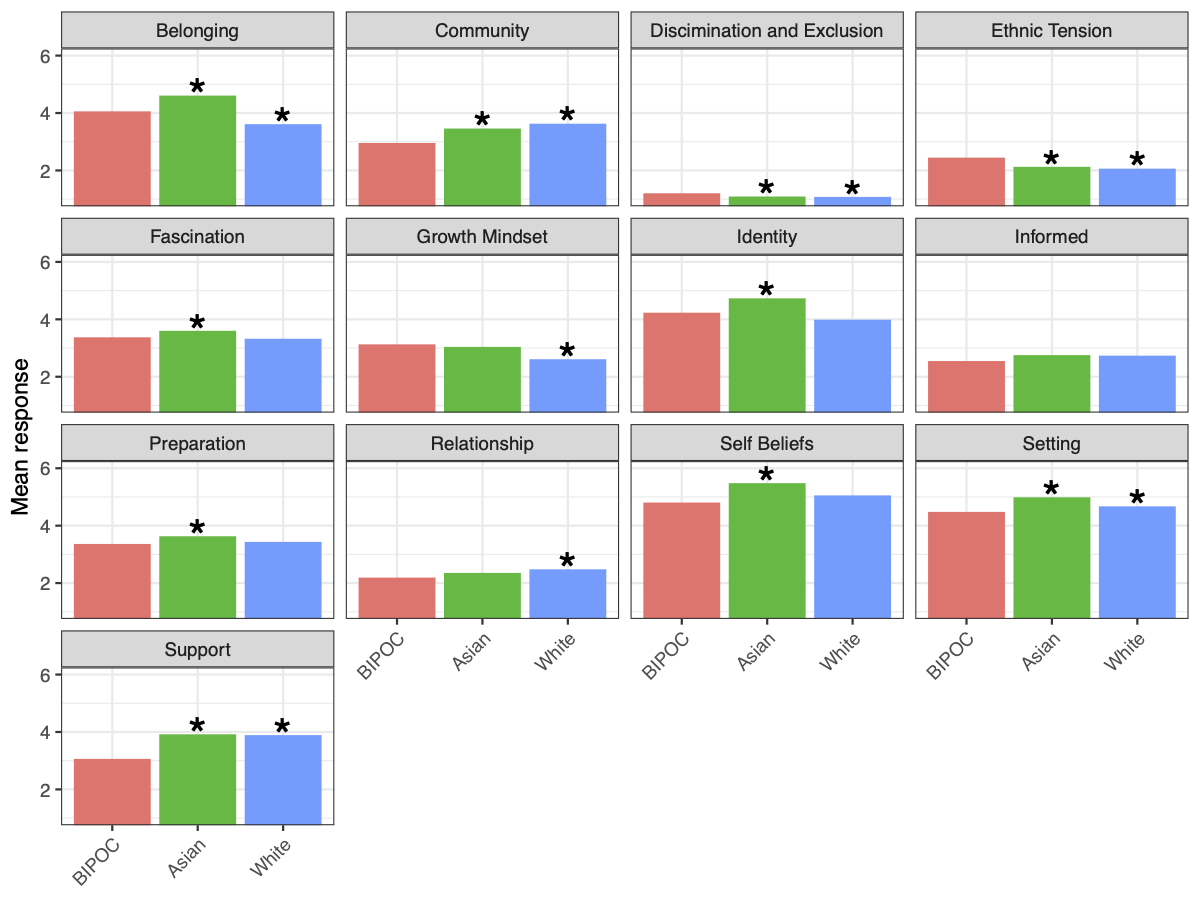


Figure A3. Mean responses from the Improving Undergraduate Student Education post-semester survey dataset for three ethnic groups BIPOC, Asian, and White from n = 286 respondents who were enrolled in the Data8: Foundations of Data Science course.

*Survey Questions and Composite Measures*

Improving Undergraduate STEM Education Post Semester Survey - Individual Differences

1. Fascination
   1. Participants read the following prompt “For each of the following statements, please indicate how important it was in your decision to take a data science course” prior to providing their agreement on a scale from 1 = Not at all important to 5 = Extremely important. A composite score was calculated by taking the average of all items (𝞪 = .78; BIPOC: *M* = 3.37, *SD* = 1.32; Asian: *M* = 3.60, *SD* = 1.29; White: *M* = 3.32, *SD* = 1.38) such that lower scores indicate less fascination and higher scores indicate greater fascination.
      1. Data science meets a requirement for my major.
      2. Data science skills are important to my major.
      3. Data science skills will improve my chances of getting a good job after graduation.
      4. Data science skills are important to my extra-curricular activities.
      5. Data science skills will help me make an impact and solve problems in society.
      6. Data science is intellectually interesting to me.
      7. Data literacy is important for everyone these days no matter what their career or major.
2. Growth Mindset
   1. Participants completed the version of the growth mindset scale that was used in Cavanagh et al. (2018) in which they read each of the following statements and selected the degree to which they disagree or agree with each statement on a scale from 1 = “strongly disagree” to 6 = “strongly agree.” Each item was reverse-scored and a composite score was calculated by taking the average of all items (𝞪 = .85; BIPOC: *M* = 3.13, *SD* = 1.72; Asian: *M* = 3.04, *SD* = 1.66; White: *M* = 2.61, *SD* = 1.57) such that lower scores indicate a lower growth mindset and higher scores indicate a greater growth mindset.
      1. You have a certain amount of intelligence, and you can’t really do much to change it. [reverse-scored]
      2. Your intelligence is something about you that you can’t change very much. [reverse-scored]
      3. You can learn new things, but you can’t really change your basic intelligence. [reverse-scored]
      4. People in my data science class believe that certain types of people have an aptitude for data science and they can’t really do much to change it. [reverse-scored]
3. Motivated strategies for learning; Expectancy Component: Self-Efficacy for Learning and Performance (referred to as “Self Beliefs” in Figure A3)
   1. Participants read the following prompt “For the following questions, please think about the data science course you are in this semester that occupies the most time.” and then completed a subset of items from Pintrich (1991) on a scale from 1 = Not at all true of me and 7 = Very true of me. A composite score was calculated by taking the average of all items (𝞪 = .94; BIPOC: *M* = 4.80, *SD* = 1.53; Asian: *M* = 5.48, *SD* = 1.30; White: *M* = 5.05, *SD* = 1.63) such that lower scores indicate less favorable judgements of one's abilities and confidence to complete tasks whereas higher scores indicate more favorable judgements of one’s abilities and confidence to complete tasks.
      1. I believe I will receive an excellent grade in this class.
      2. I’m certain I can understand the most difficult material presented in the readings for this course.
      3. I’m confident I can learn the basic concepts taught in this course.
      4. I’m confident I can understand the most complex material presented by the instructor in this course.
      5. I’m confident I can do an excellent job on the assignments and tests in this course.
      6. I’m certain I can master the skills being taught in this class.
      7. I’m confident I can find the information I need to overcome programming challenges.
4. Identity
   1. Participants read the following prompt three statements that were developed based on past research (Chemers et al., 2011; Hazari et al., 2013; & Trujillo & Tanner, 2014) and sought to assess their scientific identity. Each statement was rated on a scale from 1 = No, not at all and 6 = Yes, very much. A composite score was calculated by taking the average of all items (𝞪 = .84; BIPOC: *M* = 4.23, *SD* = 1.84; Asian: *M* = 4.73, *SD* = 1.66; White: *M* = 3.99, *SD* = 1.88) such that lower scores indicate less identification with data science whereas higher scores indicate more identification with data science.
      1. I see myself as a data science person.
      2. I could imagine myself doing data science in the future.
      3. In general, being good at data science is an important part of my self-image.
5. Belonging
   1. Participants read the following three statements that have been used in past research (Trujillo & Tanner, 2014) to assess the extent to which they feel that they belong in the campus community on a scale from 0 = Strongly disagree to 10 = Strongly agree. A composite score was calculated by taking the average of all items (𝞪 = .97; BIPOC: *M* = 4.06, *SD* = 1.64; Asian: *M* = 4.61, *SD* = 1.36; White: *M* = 3.61, *SD* = 1.71) such that lower scores indicate less perceptions of belonging in the campus community whereas higher scores indicate greater perceptions of belonging in the campus community.
      1. I see myself as a part of the campus community.
      2. I feel that I am a member of the campus community.
      3. I feel a sense of belonging to the campus community.
6. Experienced Discrimination
   1. Participants completed a version of the Experienced Discrimination-Exclusion scale (Hurtado & Carter, 1997) that was tailored to reflect participants’ experiences of discrimination based on their race/ethnicity in their data science class. Participants read the following prompt “Please describe your experiences with discrimination and/or exclusion.” and then rated their agreement with each of the following statements on a scale from 1 = Not at all to 3 = Frequently. A composite score was calculated by taking the average of all items (𝞪 = .85; BIPOC: *M* = 1.20, *SD* = .46; Asian: *M* = 1.09, *SD* = .31; White: *M* = 1.08, *SD* = 0.27) such that lower scores indicate less experiences of discrimination based on racial/ethnicity in their data science class while higher scores indicate greater experiences of discrimination based on racial/ethnicity in their data science class.
      1. I have been insulted or threatened by other students in a data science class because of my race/ethnicity.
      2. I have heard uGSIs in a data science class make inappropriate remarks regarding minorities.
      3. I have heard instructors in a data science class make inappropriate remarks regarding minorities.
      4. I have felt excluded from activities in a data science class because of my race/ethnicity.
7. Perceptions of campus racial-climate tension
   1. Participants completed a version of the Perceptions of Campus Racial-Ethnic Tension scale (Hurtado & Carter, 1997) that was tailored to reflect participants’ experiences of racial/ethnic conflict in their data science class. Participants read the following prompt “Please share your perceptions of campus racial-ethnic tension.” and then rated their agreement with each of the following statements on a scale from 0 = Strongly disagree to 3 = Strongly agree. After reverse-scoring the second item, a composite score was calculated by taking the average of all items (𝞪 = .64; BIPOC: *M* = 2.45, *SD* = 1.22; Asian: *M* = 2.12, *SD* = 1.16; White: *M* = 2.06, *SD* = 1.09) such that lower scores indicate less of experiences of racial/ethnic conflict in their data science class while higher scores indicate greater experiences of racial/ethnic conflict in their data science class.
   2. 0 = "strongly disagree" to 4 = "strongly agree"
      1. I perceive there is a lot of racial conflict in my data science class.
      2. I perceive that students of different racial/ethnic origins communicate well with one another. [reverse-scored]
      3. I perceive there is little trust between minority student groups and instructors in my data science class.
8. Math Sense of Belonging (referred to in the “Setting” panel in Figure A3)
   1. Participants completed a version of the Math Sense of Belonging scale (Good et al., 2012) that was tailored to reflect participants' sense of belonging in a data science setting. Participants read the following prompt “When I am in a data science setting…” and then rated their agreement with each of the following statements on a scale from 1 = Strongly disagree to 8 = Strongly agree. After reverse-scoring the second item, a composite score was calculated by taking the average of all items (𝞪 = .96; BIPOC: *M* = 4.48, *SD* = 1.70; Asian: *M* = 4.99, *SD* = 1.38; White: *M* = 4.67, *SD* = 1.54) such that lower scores indicate a lower sense of belonging in data science while higher scores indicate greater sense of belonging in data science.
      1. I feel that I belong in the data science community
      2. I consider myself a member of the data science world.
      3. I feel like I am part of the data science community.
      4. I feel a connection with the data science community.
      5. I feel like an outsider.
      6. I feel accepted.
      7. I feel respected.
      8. I feel disregarded. [reverse-scored]
      9. I feel valued.
      10. I feel neglected. [reverse-scored]
      11. I feel appreciated.
      12. I feel excluded. [reverse-scored]
      13. I feel like I fit in.
      14. I feel insignificant. [reverse-scored]
      15. I feel at ease.
      16. I feel anxious. [reverse-scored]
      17. I feel comfortable.
      18. I feel tense. [reverse-scored]
      19. I feel nervous. [reverse-scored]
      20. I feel content.
      21. I feel calm.
      22. I feel inadequate. [reverse-scored]
      23. I wish I could fade into the background and not be noticed. [reverse-scored]
      24. I try to say as little as possible. [reverse-scored]
      25. I enjoy being an active participant
      26. I wish I were invisible. [reverse-scored]
      27. I trust the testing materials to be unbiased.
      28. I have trust that I do not have to constantly prove myself.
      29. I trust my instructors to be committed to helping me learn.
      30. Even when I do poorly, I trust my instructors to have faith in my potential.
9. Science Capital
   1. To understand how participants’ perceive themselves as having science capital, the research team developed a series of subscales to assess the following dimensions of science capital; relationships with science, informed about science, and scientific forms of cultural capital (Dewitt et al., 2016).
   2. Relationship with Science
      1. Participants read the following statement and rated the degree to which they are informed about science from 1 = Not at all informed to 4 = Very well informed. Scores were derived by using participants' raw responses to this item (BIPOC: *M* = 2.19, *SD* = .59; Asian: *M* = 2.35, *SD* = .52; White: *M* = 2.48, *SD* = .58) such that lower scores indicate being less informed about science whereas higher scores indicate being more informed about science.
         1. Which of these statements best describes your relationship with science?
   3. Informed about Science
      1. Participants read the following statement and rated the degree to which they agree with it from -2 = Strongly disagree to 2 = Strongly agree. Scores were derived by using participants' raw responses to this item (BIPOC: *M* = 2.55, *SD* = .71; Asian: *M* = 2.75, *SD* = .63; White: *M* = 2.73, *SD* = .57) such that lower scores indicate being less informed about science, scientific research, and developments whereas higher scores indicate being more informed about science, scientific research, and developments.
         1. How well informed do you feel, if at all, about science, and scientific research and developments?
   4. Scientific Forms of Social Capital - Science Mentor (referred to in the “Community” panel in Figure A3)
      1. Participants read the following prompt “Consider the extent to which there has been someone in your life who has done the following for you.” and then rated the extent to which there has been someone there for them for the following statements on a scale from 1 = Not at all to 5 = To a very large extent. A composite score was calculated by taking the average of all items (𝞪 = .85; BIPOC: *M* = 2.96, *SD* = 1.30; Asian: *M* = 3.46, *SD* = 1.13; White: *M* = 3.63, *SD* = 1.07) such that lower scores indicate a lower extent of having a science mentor while higher scores indicate greater extent of having a science mentor.
         1. Given me the impression that they believe in me being able to do science.
         2. Gone out of their way to promote my interest in science.
         3. Helped me figure out for myself how to answer a research question.
         4. Taught me specific research or analysis skills.
   5. Scientific Forms of Social Capital - Social Support (referred to in the “Support” panel of Figure A3)
      1. Participants read the following prompt “How much do you agree with the following statements?” and then rated their agreement with the following statements on a scale from -2 = Strongly disagree to 2 = Strongly agree. A composite score was calculated by taking the average of all items (𝞪 = .85; BIPOC: *M* = 3.06, *SD* = 1.51; Asian: *M* = 3.92, *SD* = 1.15; White: *M* = 3.89, *SD* = 1.30) such that lower scores indicate a lower social support while higher scores indicate greater social support.
         1. I have friends who take science courses or are going to major in a scientific field.
         2. One or both of my parents think science is very interesting.
         3. People in my family work as scientists or use science in their jobs.
         4. People in my community at home work as scientists or use science in their jobs.
         5. I have someone I trust for career advice in my field of science.
         6. I have someone I trust for academic advice in my field of science.
   6. Scientific Forms of Cultural Capital (referred to in the “Preparation” panel of Figure A3)
      1. Participants read the following prompt “How much do you agree with the following statements?” and then rated the degree to which they agree with the following statements on a scale from -2 = Strongly disagree to 2 = Strongly agree. A composite score was calculated by taking the average of all items (𝞪 = .90; BIPOC: *M* = 3.36, *SD* = 1.06; Asian: *M* = 3.63, *SD* = .98; White: *M* = 3.43, *SD* = 1.12) such that lower scores indicate a lower scientific forms of cultural capital while higher scores indicate greater scientific forms of cultural capital.
         1. I have a good understanding of what courses to take to prepare myself for a career in my field of science.
         2. I have a good understanding of what extracurricular activities will prepare me for a career in my field of science.
         3. I have a good understanding of how scientists in my field do their work.
         4. I have a good understanding of what to do next to improve my skills in my field of science.
         5. I have a good understanding of the types of jobs after graduation that will help me get a good start in my field of science.
         6. I have a good understanding of the skills that are valued in my field of science.

My Experience Survey - Individual Differences Measures

1. Climate
   1. Participants read the following prompt “Please indicate how much you agree with the following statements:” prior to providing their agreement on a scale from 1 = Not at all important to 5 = Extremely important. A composite score was calculated by taking the average of all items (𝞪 = .83; BIPOC: *M* = 4.47, *SD* = 1.27; Asian: *M* = 4.69, *SD* = 1.06; White: *M* = 4.79, *SD* = 1.07) such that lower scores indicate both less comfort on campus and campus values diversity, equity, and inclusion whereas higher scores indicate both greater comfort on campus and campus values diversity, equity, and inclusion .
      1. Overall, I am comfortable with the climate at UC Berkeley.
      2. Overall, I am comfortable with the climate in my [department/program/unit].
      3. Overall, I am comfortable with the climate in my classes.
      4. Diversity, equity, and inclusion are important to me.
      5. Diversity, equity, and inclusion are values promoted in my [department/program/unit].
      6. Diversity, equity, and inclusion are values promoted at UC Berkeley.
2. Exclusion
   1. Participants read the prompt “Within the past year, how often have you personally experienced any of the following exclusionary, bullying, or intimidating behaviors AT UC BERKELEY?” for the first two statements and the following prompt “Within the past 12 months, how frequently have you personally experienced any of the following behaviors at UC Berkeley…” for the last statement prior to providing their frequency of experiencing these behaviors on a scale from 1 = Never to 6 = Very often. A composite score was calculated by taking the average of all items (𝞪 = .72; BIPOC: *M* = 5.25, *SD* = 1.14; Asian: *M* = 5.47, *SD* = .89; White: *M* = 5.44, *SD* = .99) such that lower scores indicate less frequently experiencing exclusionary, bullying, or intimidating behaviors whereas higher scores indicate more frequently experiencing exclusionary, bullying, or intimidating behaviors.
      1. I have been regularly teased or was the brunt of pranks or practical jokes.
      2. I experienced inappropriate comments about my appearance, way of speaking, lifestyle, family, or culture.
      3. ...demands/threats.
3. Institution
   1. Participants read the prompt “Please indicate your agreement with the following statements:” for the first statement and the following prompt “Overall, the CHANCELLOR, VICE CHANCELLORS, DEANS, AND OTHER LEADERSHIP STAFF at UC Berkeley...” for the remaining three statements prior to providing their agreement on a scale from 1 = Strongly disagree to 6 = Strongly agree. A composite score was calculated by taking the average of all items (𝞪 = .89; BIPOC: *M* = 3.90, *SD* = 1.35; Asian: *M* = 4.29, *SD* = 1.28; White: *M* = 4.25, *SD* = 1.26) such that lower scores indicate less feelings of institutional trust whereas higher scores indicate greater feelings of institutional trust.
      1. I feel valued by faculty in the classroom/learning environment.
      2. Deal with me honestly and ethically.
      3. Show concern for people's rights.
      4. Can be trusted to have my best interests in mind.
4. Respect
   1. Participants read the prompt “Please indicate your agreement with the following statements” prior to providing their agreement on a scale from 1 = Strongly disagree to 6 = Strongly agree. A composite score was calculated by taking the average of all items (𝞪 = .85; BIPOC: *M* = 4.68, *SD* = 1.28; Asian: *M* = 5.02, *SD* = .89; White: *M* = 5.10, *SD* = 1.11) such that lower scores indicate less feelings of respect whereas higher scores indicate greater feelings of respect.
      1. [POPFILL] of my race/ethnicity are respected on this campus.
      2. [POPFILL] of my socio-economic status are respected on this campus.
      3. [POPFILL] of my gender or gender identity are respected on this campus.
      4. [POPFILL] of my age are respected on this campus.
      5. [POPFILL] of my religious beliefs are respected on this campus.
      6. [POPFILL] of my incarceration background are respected on this campus.

**Appendix References**

Cavanagh, A. J., Chen, X., Bathgate, M., Frederick, J., Hanauer, D. I., & Graham, M. J. (2018). Trust, Growth Mindset, and Student Commitment to Active Learning in a College Science Course. CBE life sciences education, 17(1), ar10.

Chemers, M. M., Zurbriggen, E. L., Syed, M., Goza, B. K., & Bearman, S. (2011). The role of efficacy and identity in science career commitment among underrepresented minority students. Journal of Social Issues, 67(3), 469–491.

Dewitt, J., Archer, L., & Mau, A. (2016). Dimensions of science capital: exploring its potential for understanding students’ science participation. International Journal of Science Education, 38, 2431 - 2449.

Good, C., Rattan, A., & Dweck, C. S. (2012). Why do women opt out? Sense of belonging and women's representation in mathematics. Journal of Personality and Social Psychology, 102(4), 700–717.

Hazari, Z., Sadler, P. M., & Sonnert, G. (2013). The science identity of college students: Exploring the intersection of gender, race, and ethnicity. Journal of College Science Teaching, 42(5), 82-91.

Hurtado, S., & Carter, D. F. (1997). Effects of college transition and perceptions of the campus racial climate on Latino college students' sense of belonging. Sociology of Education, 70(4), 324–345.

Miles, M. B., & Huberman, A. M. (1994). *Qualitative data analysis: An expanded sourcebook*. sage.

Moore, D. W., Bathgate, M. E., Chung, J., & Cannady, M. A. (2011). Technical report: Measuring activation and engagement. *Activation Lab, Enables Success Study*.

Pintrich, P. R. (1991). A manual for the use of the Motivated Strategies for Learning Questionnaire (MSLQ).

Trujillo, G., Aguinaldo, P. G., Anderson, C., Bustamante, J., Gelsinger, D. R., Pastor, M. J., ... & Riggs, B. (2015). Near-peer STEM mentoring offers unexpected benefits for mentors from traditionally underrepresented backgrounds. *Perspectives on undergraduate research and mentoring: PURM*, *4*(1).

Yin, R. K. (2009). *Case study research: Design and methods* (Vol. 5). sage.
